# Lion Hearts: applying implementation mapping to plan the delivery of a family-based physical activity intervention in community CrossFit affiliates

**DOI:** 10.3389/fpubh.2026.1970725

**Published:** 2026-10-06

**Authors:** Janette Watkins, Janelle Goss, Kelton Mehls, Ali Marshall, Deirdre Dlugonski, Danielle Symons Downs

**Affiliations:** 1 Department of Kinesiology, College of Health and Human Development, The Pennsylvania State University, University Park, PA, United States; 2 Department of Health and Human Development, University of Pittsburgh, Pittsburgh, PA, United States; 3 Department of Health & Clinical Sciences, College of Health Sciences, University of Kentucky, Lexington, KY, United States; 4 Department of Obstetrics and Gynecology, College of Medicine, The Pennsylvania State University, University Park, PA, United States

**Keywords:** community fitness, family-based intervention, implementation mapping, implementation strategies, maternal health, physical activity

## Abstract

**Background:**

Community-based physical activity interventions face challenges in adoption, implementation, and sustainability. Lion Hearts is a family-based physical activity intervention designed to promote cardiovascular health among mothers and children aged 3–6 years. This study applied Implementation Mapping during intervention development to identify implementation determinants and develop stakeholder-informed strategies for subsequent pilot testing.

**Methods:**

The five iterative tasks of Implementation Mapping were applied through a co-design process with seven CrossFit affiliate owner-coaches: (1) conducting an implementation needs and assets assessment and identifying adopters and implementers; (2) specifying implementation outcomes, performance objectives, determinants, and change objectives; (3) selecting theoretical change methods and implementation strategies; (4) producing implementation protocols and materials; and (5) developing a prospective implementation-evaluation plan. Two 60-min co-design sessions were conducted approximately eight weeks apart. The first elicited barriers and facilitators to adoption and delivery within affiliate settings. Audio recordings were transcribed verbatim, and three researchers reviewed and coded both transcripts using structured matrices, supplemented by facilitator notes. Findings were synthesized using rapid qualitative analysis and integrated with behavioral theory and implementation science frameworks to identify implementation determinants and develop implementation strategies and materials. This study applied the five tasks of Implementation Mapping to identify implementation determinants and develop stakeholder-informed strategies, delivery supports, and a prospective evaluation plan for subsequent pilot testing.

**Results:**

Stakeholders identified implementation determinants related to coach knowledge and self-efficacy, participant engagement and competing demands, class structure and flow, scheduling, staffing, equipment availability, and compatibility with affiliate workflows. These findings informed implementation strategies and delivery supports targeting affiliate owners and coaches, including coach training resources, structured session templates, class-flow guidance, and fidelity-monitoring tools. Performance objectives and an implementation logic model were developed to specify hypothesized pathways linking determinants and strategies to prospective implementation outcomes. An evaluation plan was developed to assess reach, adoption, feasibility, acceptability, fidelity, and maintenance during subsequent pilot implementation.

**Conclusion:**

Implementation Mapping and stakeholder co-design produced a theory-informed, contextually responsive implementation package for Lion Hearts. Although implementation outcomes have not yet been evaluated, the resulting strategies, materials, and prospective evaluation plan are positioned for testing and refinement in community fitness settings.

**Clinical trial registration:**

ClinicalTrials.gov, NCT07147387.

## 1 Introduction

Cardiovascular disease (CVD) remains the leading cause of morbidity and mortality among women in the United States, accounting for approximately one in every five female deaths (1). Despite widespread public health efforts to promote physical activity as a preventive strategy to reduce cardiovascular disease, many women, particularly mothers of young children, experience substantial barriers to engaging in regular physical activity. These barriers include time constraints, caregiving responsibilities, fatigue, and limited access to supportive environments (2). These challenges contribute to persistently low levels of moderate-to-vigorous physical activity (3), increasing women’s long-term risk for cardiometabolic disease.

Importantly, strength training remains especially low among women despite that it is important for women’s cardiovascular and metabolic health (4, 5). There is growing evidence that resistance training independently reduces cardiovascular disease risk, improves metabolic health, preserves functional capacity, and supports healthy aging (6–8). However, fewer than 27% of U. S. women meet the national guidelines for muscle-strengthening, versus with about 35% of men (9). This discrepancy is even larger among mothers of young children, who often face barriers of fatigue, time constraints, and lack of access to supportive environments (e.g., social support, childcare) (10, 11). Given the low rates of adoption and many benefits of strength/resistance exercise, interventions that simultaneously promote aerobic activity and muscle-strengthening exercises may offer a particularly promising strategy for improving women’s cardiovascular health.

Importantly, the implications of maternal physical inactivity extend beyond individual health. Maternal and child physical activity levels are positively associated, including among mothers and preschool-aged children. These associations suggest that supporting maternal physical activity may also create opportunities to promote activity among children (12). Indeed, children of physically active mothers are significantly more likely to meet physical activity guidelines, while maternal inactivity is associated with lower activity levels, reduced fitness, and elevated cardiometabolic risk in offspring (13–15). These intergenerational associations highlight a critical opportunity for prevention: targeting maternal physical activity may yield dual benefits by improving health outcomes for both mothers and their children.

Childhood represents a foundational period for establishing lifelong health behaviors. Physical activity patterns developed during this stage tend to track into adulthood, influencing long-term cardiovascular risk and overall well-being (16, 17). However, many children do not achieve recommended levels of physical activity (18), underscoring the need for innovative, family-centered approaches that promote engagement in movement behaviors early in life. Interventions that simultaneously engage mothers and children may be particularly effective, as they leverage shared environments, reciprocal motivation, and behavioral modeling to reinforce sustained behavior change.

Family-based interventions represent one potential approach for promoting physical activity across generations. By engaging the mother–child dyad, these approaches can draw upon shared environments, parental support, behavioral modeling, and opportunities for co-participation (19–21). By targeting the mother–child dyad as the unit of change, these approaches capitalize on bidirectional influences within the family system, where parental behaviors shape child outcomes and children’s engagement can, in turn, reinforce parental adherence (22, 23). Although clinical and school-based settings can support family engagement, they may not consistently provide opportunities for mothers and children to participate together in the same physical activity sessions. Community-based fitness settings represent an innovative and scalable solution for delivering family-centered interventions. In particular, CrossFit affiliates provide an existing infrastructure that supports functional movement, social connection, and scalable programming for individuals of varying ages and abilities (6, 24). As of early 2025, CrossFit maintains over 11,000 business owners (affiliates) and more than 100,000 credentialed (25). Complementing this, the brand operates approximately 10,000 paying affiliate gyms across more than 150 countries, constituting one of the largest fitness networks globally (26). Crucially, many CrossFit coaches not only complete foundational certifications (such as the Level 1 Trainer credential)but also pursue CrossFit Kids certification, which equips them with methods to deliver safe, engaging, and developmentally appropriate fitness to youth ages 3 to 17 years (27). This dual certification framework enables affiliates to program for mothers alongside their children, effectively supporting entire families under one roof through synchronized, structured exercise offerings. These environments are uniquely positioned to facilitate co-participation between mothers and children, reducing common barriers such as childcare and scheduling while fostering social support and accountability. Leveraging such community-based platforms offers a practical pathway for embedding cardiovascular disease prevention into everyday life.

Theory-driven intervention development can strengthen an intervention’s content and hypothesized mechanisms, but it does not ensure adoption, consistent delivery, or sustainability in routine practice. These outcomes also depend on the behaviors of adopters and implementers and on the organizational and contextual conditions surrounding delivery. Implementation Mapping extends the intervention-development process by systematically identifying these determinants and developing strategies to support implementation (28). Intervention Mapping provides a systematic framework for developing, implementing, and evaluating evidence-based health-promotion interventions (29). Lion Hearts was previously developed through Intervention Mapping Steps 1–4, which addressed needs assessment, program outcomes and objectives, intervention design, and program production (30). Although implementation planning is included in Step 5 of Intervention Mapping, Implementation Mapping expands this component into a more detailed five-task process for developing and evaluating strategies that support program adoption, implementation, and sustainability (31). The five iterative Implementation Mapping tasks are to: (1) conduct an implementation needs and assets assessment and identify program adopters and implementers; (2) specify implementation outcomes, performance objectives, determinants, and change objectives; (3) select theoretical change methods and implementation strategies; (4) produce implementation protocols and materials; and (5) evaluate implementation outcomes (31). The present study applied these five tasks to develop the implementation approach for delivering Lion Hearts through community CrossFit affiliates.

Lion Hearts is a family-based physical activity intervention targeting mother–child dyads with children aged 3–6 years that was previously developed through Steps 1–4 of Intervention Mapping (30). The program integrates strength- and aerobic-based exercise, cardiovascular health education, and family-centered behavior change strategies within community CrossFit settings. Building on this foundation, the present study extends the Intervention Mapping process to implementation planning, with the goal of identifying anticipated determinants of adoption and delivery and developing stakeholder-informed implementation strategies for subsequent pilot testing in community fitness settings.

Specifically, this study aimed to: (1) assess anticipated implementation needs and assets and identify the organizational adopters and frontline implementers of Lion Hearts; (2) specify implementation outcomes, stakeholder-specific performance objectives, determinants, and corresponding change objectives; (3) select theoretical change methods and implementation strategies addressing the identified determinants; (4) develop and refine practical implementation protocols and materials through stakeholder co-design; and (5) develop a prospective plan for evaluating implementation outcomes during pilot delivery. By explicitly documenting these linkages, this study provides a structured implementation plan and testable logic model to guide the next phase of Lion Hearts evaluation.

## 2 Methods

### 2.1 Study design and relationship between frameworks

This study applied the five iterative tasks of Implementation Mapping to develop an implementation approach for Lion Hearts in community CrossFit affiliates (31). Lion Hearts itself was previously developed through Intervention Mapping Steps 1–4, including needs assessment, specification of intervention outcomes and objectives, theory-informed intervention design, and program production (30). The current study therefore focused on planning how the previously developed intervention would be adopted, delivered, and evaluated in community settings. Specifically, this focus was achieved by identifying anticipated implementation determinants, specifying stakeholder-specific implementation behaviors and performance objectives, linking determinants to theory-informed implementation strategies and hypothesized mechanisms, developing practical delivery supports, and specifying a prospective evaluation plan for subsequent pilot testing.

Consistent with community-based participatory research, this phase emphasized stakeholder engagement to ensure that implementation planning reflected the practical considerations of community-based delivery (32, 33). A co-design approach was used to integrate the expertise of experienced CrossFit affiliate owner-coaches, who represented both organizational adopters and frontline implementers, into the identification of implementation determinants and the development and refinement of implementation strategies and delivery supports.

### 2.2 Participants and setting

Seven CrossFit affiliate owner-coaches were purposively recruited from local affiliate settings to participate in the implementation planning process. Eligibility criteria required that participants be both certified CrossFit coaches and affiliate owners, with a minimum of five years of affiliate operation. This criterion was used to ensure that participants possessed substantial experience not only in delivering CrossFit programming but also in managing the logistical, financial, and community-related demands of sustaining an affiliate. Participants were also required to have experience leading group-based training sessions and adapting workouts for individuals with varying fitness levels and abilities. CrossFit coaching credentials ranged from Level 2 to Level 4.

This purposive sampling approach was selected to engage a developmental stakeholder panel with direct, practice-based expertise relevant to both organizational adoption and frontline delivery within real-world CrossFit settings. In their roles as affiliate owners, participants provided perspectives on organizational considerations such as staffing, scheduling, resources, and program integration; in their roles as coaches, they provided perspectives on session delivery, movement adaptation, participant engagement, and class management. Their dual roles therefore provided input into both adopter- and implementer-level considerations during this formative planning phase.

The stakeholder panel was intentionally small because its purpose was to generate practice-based input for iterative implementation planning rather than to achieve thematic saturation, characterize determinants across the broader CrossFit affiliate population, or evaluate implementation outcomes. Implementation Mapping is an iterative, participatory planning approach that incorporates stakeholder expertise into the identification of implementation needs and development of contextually relevant strategies (34). Consistent with co-design methodologies, small groups of key stakeholders can support iterative identification of implementation barriers and facilitators and refinement of intervention and implementation materials (35). Accordingly, findings from this panel were treated as stakeholder-informed design inputs rather than as a comprehensive assessment of implementation determinants across CrossFit affiliates.

CrossFit affiliates served as the intended implementation setting for Lion Hearts. These environments are characterized by group-based training, scalable programming, and established coaching infrastructure, providing a potential setting for delivering family-based interventions. However, variability in class structure, scheduling, staffing, space, equipment, and organizational workflows informed the need for implementation strategies that could accommodate contextual variation while preserving core intervention components.

### 2.3 Implementation mapping framework and theoretical foundations

Lion Hearts was previously developed using Steps 1–4 of Intervention Mapping, which focused on identifying health and behavioral needs, specifying intervention objectives, selecting theory-informed behavior-change methods, and developing the intervention components. The present study focused specifically on implementation planning and applied the five tasks of Implementation Mapping: (1) conducting an implementation needs and assets assessment and identifying adopters and implementers; (2) specifying implementation outcomes, performance objectives, determinants, and change objectives; (3) selecting theoretical change methods and designing implementation strategies; (4) producing and refining implementation protocols and materials; and (5) developing a prospective plan to evaluate implementation outcomes. Figure 1 summarizes the study procedures and outputs associated with each task.

**Figure 1:**
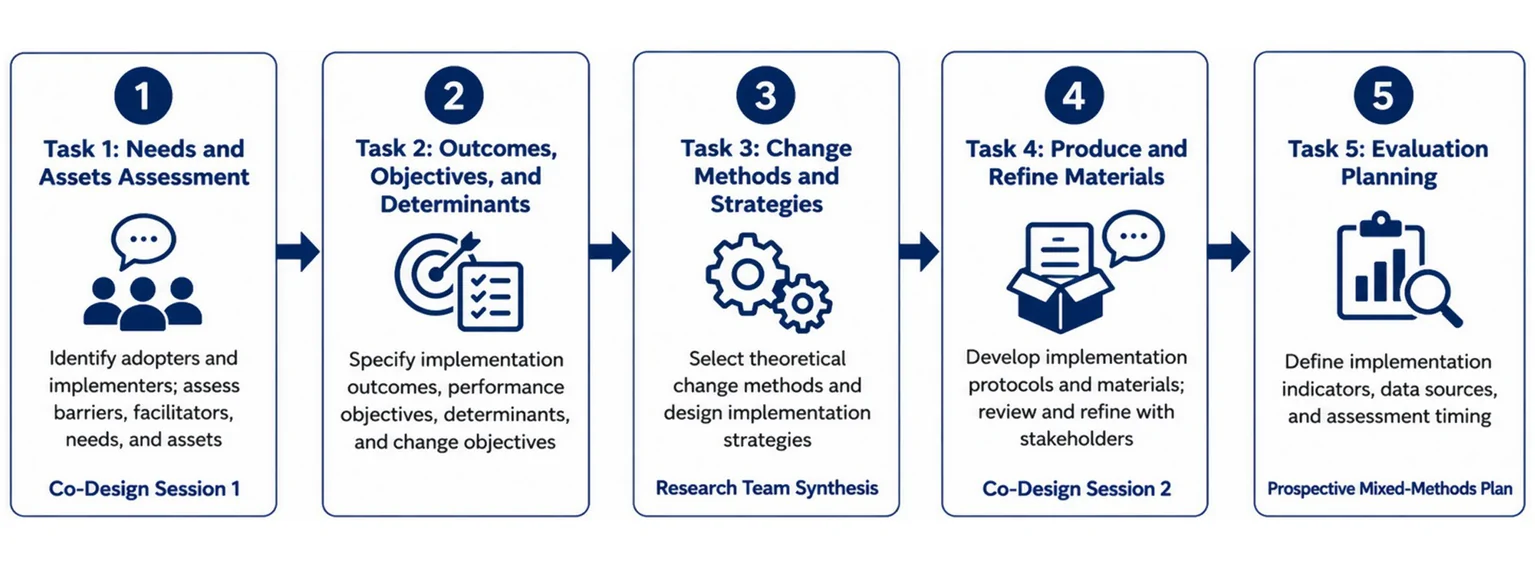
Implementation mapping process and objectives.

Intervention-level and implementation-level mechanisms were considered separately. At the intervention level, Self-Determination Theory (SDT) (36) and Social Cognitive Theory (SCT) (37) informed the hypothesized mechanisms through which Lion Hearts is expected to influence mother and child behavioral outcomes (Figure 2). Specifically, Lion Hearts seeks to enhance autonomy, competence, and relatedness through shared mother–child participation, skill development, goal setting, and social support. Consistent with SCT, the intervention also leverages observational learning, self-efficacy, behavioral capability, and parental modeling to promote sustained engagement in physical activity. Together, these theories describe the hypothesized pathways through which participation in Lion Hearts may influence family physical activity behaviors.

**Figure 2:**
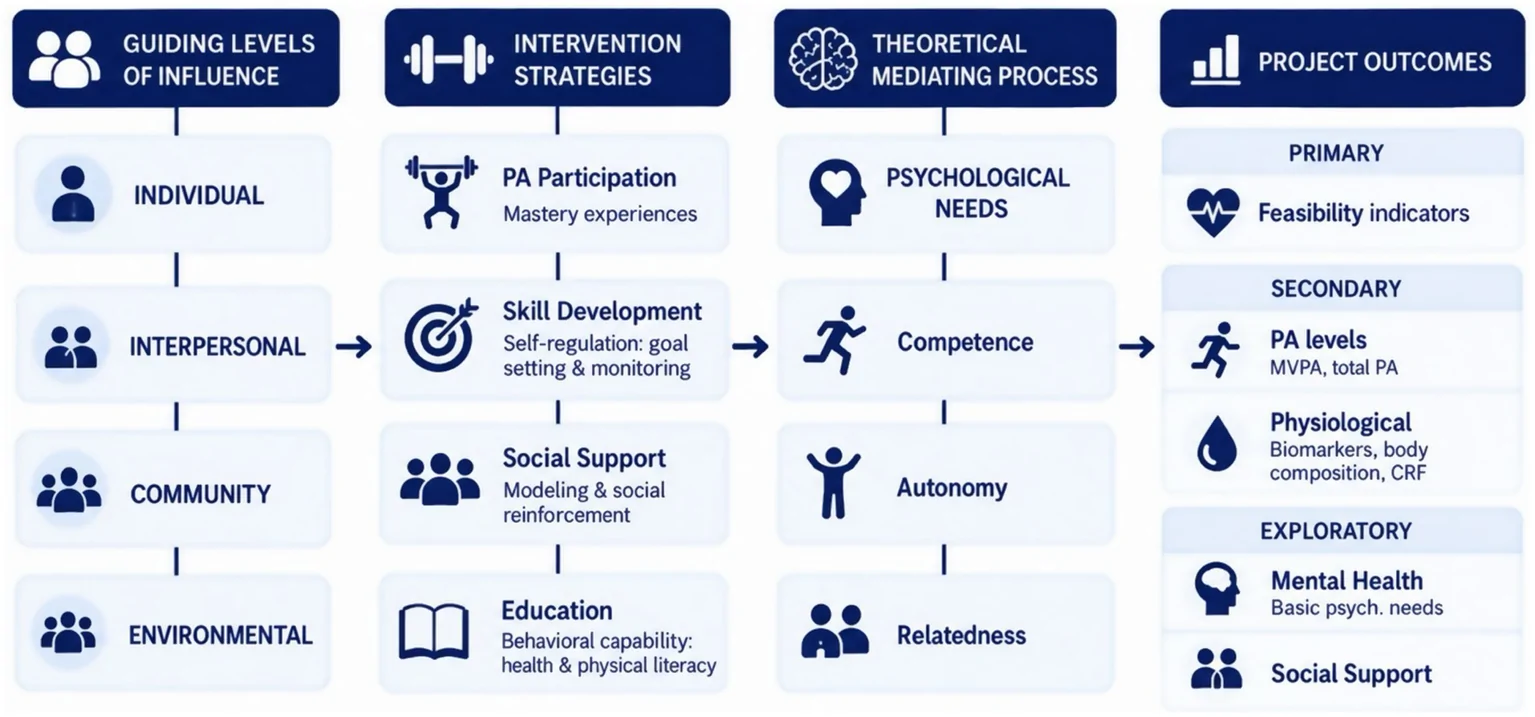
Lion hearts conceptual framework.

In contrast, implementation-level mechanisms describe how the implementation strategies are hypothesized to influence adopter and implementer behaviors, such as by increasing coach knowledge and self-efficacy, reducing perceived complexity, or improving compatibility with affiliate workflows. These implementation-level linkages were specified prospectively and are intended for empirical evaluation during subsequent pilot implementation.

### 2.4 Stakeholder recruitment and characteristics

Affiliate owner-coaches were purposively recruited from CrossFit affiliates located across the United States. Potential participants were identified through the research team’s professional networks and publicly available affiliate websites. Individuals were eligible if they were aged 18 years or older, currently worked as a CrossFit coach, and had experience owning or operating a CrossFit affiliate. Prior CrossFit Kids certification was not required because the study sought perspectives from coaches with varied experiences in youth and family-based programming.

Ten potentially eligible coaches were contacted by email using contact information obtained through professional connections or affiliate websites. Seven agreed to participate, resulting in a recruitment rate of 70%. Three coaches declined because scheduling constraints prevented their participation in the co-design sessions. No individuals declined because of concerns about the study topic or procedures.

The seven participants represented geographically diverse regions of the United States, including the Southeast (*n* = 2), Mid-Atlantic (*n* = 1), Northeast (*n* = 1), West Coast (*n* = 2), and Northwest (*n* = 1). Four participants represented affiliates in urban communities, and three represented affiliates in rural communities. All participants currently owned or had previously owned a CrossFit affiliate and had between 8 and 17 years of coaching experience. Five held a CrossFit Kids credential. Regarding general CrossFit coaching credentials, two participants held a CrossFit Level 2 credential, four held a CrossFit Level 3 credential, and one held a CrossFit Level 4 credential.

All seven participants attended both co-design sessions, resulting in 100% attendance and retention across sessions. Participant and affiliate characteristics are summarized in Table 1. Affiliate membership size and additional participant demographic characteristics were not systematically collected during this formative study and therefore could not be reported.

**Table 1:** Characteristics of participating affiliate owner-coaches and their affiliates.

Characteristic	Participating stakeholders (*N* = 7)
Recruitment and participation	
Potential participants approached, *n*	10
Enrolled, *n* (%)	7 (70%)
Declined, *n* (%)	3 (30%)
Reason for declining	Scheduling constraints
Attended Co-Design Session 1, *n* (%)	7 (100%)
Attended Co-Design Session 2, *n* (%)	7 (100%)
Attended both sessions, *n* (%)	7 (100%)
Professional experience	
CrossFit coaching experience, range in years	8–17
Current or former affiliate owners, *n* (%)	7 (100%)
CrossFit Kids credential, *n* (%)	5 (71%)
Highest CrossFit coaching credential	
Level 2, *n* (%)	2 (29%)
Level 3, *n* (%)	4 (57%)
Level 4, *n* (%)	1 (14%)
Geographical context	
Southeast, *n* (%)	2 (29%)
Mid-Atlantic, *n* (%)	1 (14%)
Northeast, *n* (%)	1 (14%)
West Coast, *n* (%)	2 (29%)
Northwest, *n* (%)	1 (14%)
Affiliate community context	
Urban, *n* (%)	4 (57%)
Rural, *n* (%)	3 (43%)

Percentages may not total 100% because of rounding. Affiliate membership size and additional participant demographic characteristics were not systematically collected. CrossFit Kids is a credential focused on the safe and developmentally appropriate delivery of CrossFit programming to children and adolescents.

### 2.5 Co-design procedures

The sessions were facilitated by JW and JG, both of whom had prior experience conducting co-design sessions with community stakeholders to promote physical activity and applying Intervention Mapping to physical activity interventions. JW identified and contacted potential participants through publicly accessible professional CrossFit networks. Neither facilitator held a supervisory or evaluative relationship with the participants. Because members of the research team were also involved in developing Lion Hearts, the team recognized that their expectations could influence facilitation and interpretation. Structured prompts, independent transcript review, and team-based consensus were used to encourage critical feedback and reduce reliance on any single researcher’s interpretation.

Two structured co-design sessions were conducted with participating affiliate owner-coaches to incorporate practitioner knowledge into the implementation-planning process. Each session lasted approximately 60 min and was facilitated by members of the research team with expertise in behavior change and community-based intervention design. The sessions were conducted approximately eight weeks apart, allowing the research team to synthesize findings from the first session and develop preliminary implementation strategies and materials for review during the second session.

The first session primarily informed Implementation Mapping Task 1 by eliciting owner-coaches’ perspectives on the factors that could influence the adoption and delivery of a family-based program in affiliate settings. Using structured prompts, participants considered the actions required of affiliate owners and coaches to adopt and deliver Lion Hearts, as well as the factors that could facilitate or impede those actions. Participants reflected on their experiences with participant engagement, class structure, and logistical constraints, with particular attention to the needs of mothers and children participating together. The discussion addressed scheduling, movement scaling, safety, equipment use, staffing, class flow, and strategies for promoting participant engagement and retention.

Following the first session, the research team synthesized stakeholder input and used the findings to complete Implementation Mapping Tasks 2 and 3. This process included specifying performance objectives for adopters and implementers, identifying determinants of the required implementation behaviors, selecting theoretical change methods, and designing preliminary implementation strategies. The resulting strategies and supporting materials were developed as prototypes for stakeholder review rather than as finalized implementation supports.

The second session primarily informed Implementation Mapping Task 4. Owner-coaches reviewed the preliminary implementation strategies and materials in relation to the needs identified during the first session and considered their anticipated usability and compatibility with typical affiliate workflows. Through guided discussion and iterative revision, the materials were modified to better align with coaching practices, participant needs, equipment availability, safety considerations, and class-management procedures. Stakeholder recommendations were used to revise the content, format, and proposed delivery of the implementation supports and to further specify the relationships among implementation determinants, strategies, mechanisms, and intended outcomes.

Both sessions were audio-recorded and transcribed verbatim to provide a detailed record of stakeholder discussions. Facilitators also maintained session notes documenting key implementation considerations and contextual observations. Transcripts and session notes were analyzed as described in Section 2.10. Collectively, the co-design findings informed Implementation Mapping Tasks 1–4 and the prospective evaluation plan developed in Task 5.

### 2.6 Task 1: implementation needs and assets assessment

Implementation Mapping Task 1 involved identifying the organizational adopters and program implementers and assessing the needs, assets, barriers, and facilitators associated with adopting and delivering Lion Hearts. CrossFit affiliate owners were identified as the primary organizational adopters responsible for deciding whether to offer the program and for allocating the staffing, scheduling, space, equipment, and other resources required for delivery. Coaches were identified as the primary implementers responsible for preparing for and delivering the intervention’s core components.

Data from the first co-design session were used to identify anticipated implementation determinants at the coach, participant, environmental, and organizational levels. Coach-level considerations included confidence in delivering a family-based program, knowledge of appropriate movement modifications, and training and resource needs. Participant-level considerations included motivation, competing demands, varying fitness levels, and the needs of mother–child dyads. Environmental and organizational considerations included available space and equipment, staffing, class flow, scheduling, safety, and compatibility with existing affiliate workflows. Existing coaching expertise, adaptable affiliate environments, established relationships with members, and experience delivering group-based fitness programming were considered potential implementation assets.

### 2.7 Task 2: implementation outcomes, performance objectives, determinants, and change objectives

Findings from the implementation needs and assets assessment were used to complete Implementation Mapping Task 2 by specifying implementation outcomes, stakeholder-specific performance objectives, determinants, and corresponding change objectives. Performance objectives were defined as the observable actions required of affiliate owners and coaches to support successful adoption and delivery of Lion Hearts.

For affiliate owners, performance objectives included committing to program adoption, identifying appropriate coaches, allocating resources, establishing feasible schedules, promoting the program to eligible families, and integrating Lion Hearts into existing affiliate operations. For coaches, performance objectives included completing program preparation and training, delivering the core intervention components, adapting activities for mother–child dyads, maintaining safety, facilitating participant engagement, and monitoring delivery fidelity.

The barriers and facilitators identified through the co-design process were examined as determinants of these performance objectives. Change objectives were then formulated by specifying what would need to change in each determinant for adopters and implementers to achieve the corresponding performance objective. Prospective implementation outcomes included reach, adoption, acceptability, feasibility, fidelity, and maintenance. These Task 2 outputs provided the foundation for selecting theoretical change methods and implementation strategies in Task 3.

### 2.8 Task 3: selection of change methods and implementation strategies

Implementation Mapping Task 3 involved selecting theoretical change methods and translating them into practical implementation strategies designed to address the determinants and change objectives identified in Task 2. Candidate methods and strategies were considered according to their anticipated ability to influence the behaviors required of affiliate owners and coaches.

The research team developed descriptive strategy labels that reflected the specific actions and materials proposed for Lion Hearts. Candidate strategies were generated by reviewing the stakeholder-identified determinants and change objectives and selecting theoretically informed methods and practical applications expected to address them. Selection also considered stakeholder recommendations, compatibility with affiliate workflows, and anticipated feasibility in community fitness settings. Members of the research team involved in intervention development and qualitative analysis completed the mapping. Proposed links among performance objectives, determinants, change methods, strategies, mechanisms, and outcomes were reviewed during team meetings, with differences resolved through discussion and consensus. Preliminary strategies were subsequently presented to owner-coaches during the second co-design session and refined in response to their feedback. The final linkages are presented in the Implementation Mapping matrix, and Supplementary Table S2 specifies each strategy’s actor, action, action target, planned timing, dose, and intended implementation outcome.

For example, strategies addressing coach knowledge and behavioral capability included structured training, implementation guidance, and session templates. Strategies targeting coach self-efficacy included opportunities for skill development, modeling, guided practice, and feedback. Strategies addressing perceived complexity and compatibility with affiliate workflows included standardized class-flow frameworks, adaptable session plans, movement-scaling guidance, and practical tools for organizing equipment and participant transitions. Strategies intended to support participant engagement included family goal-setting resources, shared activities, and materials designed to reinforce motivation and social support. For each proposed strategy, the research team specified the targeted adopter or implementer behavior, the relevant determinant, the theoretical change method, the practical implementation strategy, the hypothesized mechanism of action, and the intended implementation outcome. These prospective linkages were documented in the Implementation Mapping matrix presented in Table 2.

**Table 2:** Traceability table.

Overarching consideration	Exemplar quotation	Stakeholder source (Session 1)	Resulting decision	Refinement (Session 2)
Balance structure with flexibility: child behavior, interruptions, differing abilities, and variable class flow	“If every kid behaves, it’s a great class […] but that’s not reality.”	Rural affiliate owner-coach, CrossFit Level 3	Develop structured but adaptable session templates and class-flow guidance	Session templates were revised to provide additional options for modifying activities and responding to interruptions or changes in class flow.
Balance structure with flexibility: perceived complexity and need for adaptable programming	“You do not need strict programming—you need a flexible framework.”	Rural affiliate owner-coach, CrossFit Level 2	Avoid overly prescriptive programming and distinguish core components from adaptable elements	Materials were revised to clarify which components should remain consistent and which could be adapted based on participant needs, space, equipment, and class conditions.
Coach capability and fit: coach self-efficacy, creativity, and ability to adapt activities	“You need the right coach […] someone who can pivot and be creative.”	Urban affiliate owner-coach, CrossFit Level 3	Develop coach-selection guidance, training materials, and examples of real-time adaptations	Coach-facing materials were expanded to include movement-scaling examples, class-management guidance, and strategies for adapting activities while maintaining core components.
Coach capability and fit: child engagement and developmentally appropriate delivery	“For kids, it has to be fun first.”	Urban affiliate owner-coach, CrossFit Level 3	Prioritize enjoyment and developmentally appropriate activities	Session templates and coach guidance were revised to emphasize play-based activities, participant choice, and age-appropriate engagement strategies.
Integration within affiliate operations: scheduling, staffing, space, equipment, and local compatibility	“This only works if it fits the gym’s space, schedule, and people.”	Urban affiliate owner-coach, CrossFit Level 4	Develop affiliate-readiness, scheduling, staffing, space, and equipment-planning resources	Planning materials were revised to include prompts for adapting delivery to local schedules, staffing capacity, available space, equipment, and member characteristics.

Bracketed ellipses indicate that words were omitted from quotations for concision without changing their intended meaning. Sources are described by affiliate context and coaching credential rather than individual participant identifiers.

### 2.9 Task 4: development and refinement of implementation materials

Implementation Mapping Task 4 involved translating the strategies selected in Task 3 into preliminary implementation protocols, resources, and delivery supports. Draft materials included coach implementation guides, structured session templates for family-based classes, movement-scaling and safety guidance, fidelity-monitoring tools, class-flow frameworks, family workbooks, and goal-setting resources.

The preliminary materials were reviewed by affiliate owner-coaches during the second co-design session. Participants considered whether the materials were understandable, usable, and compatible with typical affiliate workflows and whether they adequately addressed the implementation needs identified during the first session. Feedback focused on coaching practices, equipment availability, staffing, class organization, movement modifications, safety, participant engagement, and the practical needs of mothers and children participating together.

Following the second session, the research team revised the content, format, and proposed delivery procedures of the implementation materials. Corresponding pathways in the Implementation Mapping matrix were also refined when stakeholder feedback clarified the relationships among determinants, strategies, mechanisms, and intended outcomes. The resulting implementation package represents a stakeholder-informed set of strategies and delivery supports developed for prospective testing and further refinement during pilot implementation.

### 2.10 Task 5: prospective implementation evaluation plan

Implementation Mapping Task 5 involved developing a prospective mixed-methods plan to evaluate the implementation of Lion Hearts during subsequent pilot testing. The evaluation plan was designed to assess whether the proposed implementation strategies are feasible, acceptable, and delivered as intended and to examine implementation across participating affiliates.

Implementation outcomes were specified using the RE-AIM framework (38) and included reach, adoption, implementation fidelity, and maintenance potential. Acceptability and feasibility were included as complementary implementation outcomes consistent with Proctor et al.’s implementation-outcomes taxonomy (39). Acceptability and feasibility will be assessed using the Acceptability of Intervention Measure (AIM), Intervention Appropriateness Measure (IAM), and Feasibility of Intervention Measure (FIM) respectively (40). For each outcome, prospective operational definitions, indicators, data sources, assessment time points, and criteria for successful implementation were specified.

Quantitative indicators will include participant enrollment, attendance, retention, affiliate adoption, and adherence to the intervention’s core components and implementation cost. Reach will be calculated as the number of eligible mother–child dyads enrolled divided by the number identified as eligible through screening. Adoption will be calculated at the setting and implementer levels as the number of participating affiliates and coaches divided by the respective numbers approached or invited. Representativeness will be examined by comparing participants, affiliates, and coaches with nonparticipants on available demographic, geographic, and organizational characteristics. Proposed criteria for successful implementation include mean acceptability and feasibility scores of at least 4 on a 1–5 scale and delivery of at least 80% of core intervention components. Implementation costs, including training, personnel time, materials, equipment, and facility resources, will be tracked descriptively. These indicators will be complemented by qualitative data capturing affiliate owner, coach, and participant experiences with program delivery; barriers and facilitators; and perceived fit within affiliate workflows. Because longer-term institutionalization will not be assessed during the pilot, maintenance potential will reflect intentions and perceived capacity to continue delivery. The proposed measures will also support preliminary examination of whether the implementation mechanisms specified through Tasks 2 and 3 correspond with the intended implementation outcomes. Health-related outcomes, including physical activity and cardiovascular indicators, will be assessed separately as intervention outcomes during subsequent pilot testing, consistent with the broader Lion Hearts evaluation framework. Maintaining this distinction will help determine whether observed findings reflect the effects of Lion Hearts itself, the success of its implementation, or both.

### 2.11 Qualitative analysis and integration

Data from the co-design sessions were synthesized using a rapid qualitative analysis approach (41). The analysis was hybrid deductive–inductive. Deductive categories were informed by the structured session prompts and Implementation Mapping domains, while inductive coding allowed additional concepts to emerge from stakeholder discussions. Audio recordings from both sessions were transcribed verbatim, and transcripts served as the primary data source, supplemented by facilitator notes.

Before coding, the research team developed a structured Microsoft Excel matrix to capture anticipated barriers, facilitators, implementation needs, stakeholder recommendations, and proposed delivery supports. The unit of coding was a meaningful segment of text expressing a distinct implementation-related idea, which could include one or more sentences. Three researchers independently reviewed both transcripts and coded relevant excerpts. Initial findings were organized into coach, participant, environmental, and organizational categories, with additional categories added as needed. Differences in coding or interpretation were resolved through discussion and consensus.

Recurring and conceptually related categories were grouped into the three overarching implementation considerations reported in the Results. Analytic decisions, including revisions to category labels and consensus interpretations, were documented in the master Excel matrix and research-team notes. Findings were subsequently mapped to performance objectives, theoretical change methods, implementation strategies, hypothesized mechanisms, and prospective implementation outcomes. These linkages were documented in the Implementation Mapping matrix. The structured prompts used during both sessions are provided in Supplementary File 1. Reporting was informed by relevant COREQ domains addressing the research team, study design, analysis, and interpretation (42).

### 2.12 Ethical considerations

This study was conducted as part of the broader Lion Hearts research program and was approved by the Pennsylvania State University Institutional Review Board (#00028778). All participants provided informed consent prior to participation in co-design sessions.

## 3 Results

The application of Implementation Mapping generated five related sets of outputs: (1) an assessment of implementation needs, assets, barriers, and facilitators; (2) stakeholder-specific implementation outcomes, performance objectives, determinants, and change objectives; (3) theoretically informed implementation strategies and hypothesized mechanisms; (4) a stakeholder-refined package of implementation materials and delivery supports; and (5) a prospective plan for evaluating implementation during subsequent pilot testing. The findings corresponding to each task are presented below.

### 3.1 Stakeholder participation and characteristics

Seven of the 10 affiliate owner-coaches approached agreed to participate in the study, corresponding to a recruitment rate of 70%. The three individuals who declined reported scheduling conflicts that prevented participation in the co-design sessions. All seven enrolled stakeholders attended both sessions, resulting in 100% attendance and retention.

Participants represented CrossFit affiliates across five regions of the United States: the Southeast (*n* = 2), Mid-Atlantic (*n* = 1), Northeast (*n* = 1), West Coast (*n* = 2), and Northwest (*n* = 1). Four participants represented urban affiliate settings, and three represented rural settings.

Participants had between 8 and 17 years of CrossFit coaching experience, and all currently owned or had previously owned an affiliate. Five participants held a CrossFit Kids credential. Two participants held a CrossFit Level 2 credential, four held a CrossFit Level 3 credential, and one held a CrossFit Level 4 credential. Additional stakeholder and affiliate characteristics are presented in Table 1.

### 3.2 Task 1: implementation needs and assets assessment

CrossFit affiliate owners were identified as the primary organizational adopters responsible for deciding whether to offer Lion Hearts and allocating the staffing, scheduling, space, equipment, and other resources required for delivery. Coaches were identified as the primary implementers responsible for preparing for and delivering the intervention’s core components. Mother–child dyads were identified as intervention recipients whose recruitment, participation, and retention would contribute to program reach. Analysis of the co-design data identified three overarching considerations for implementing Lion Hearts within community CrossFit affiliates: (1) balancing program structure with flexibility, (2) ensuring coach capability and fit for mother–child delivery, and (3) integrating the program within existing affiliate operations. These considerations reflected implementation determinants at the coach, participant, environmental, and organizational levels.

First, stakeholders emphasized that successful delivery would require sufficient structure to preserve the intervention’s core components while allowing coaches to respond flexibly to the unpredictable nature of mother–child classes. Child behavior, interruptions, differences in participant abilities, and class-flow demands were identified as potential challenges to consistent delivery. As one owner-coach explained, “If every kid behaves, it’s a great class […] but that’s not reality.” Rather than highly prescriptive programming, stakeholders favored an adaptable framework that would provide coaches with clear expectations and options for responding to changing class conditions: “You don’t need strict programming—you need a flexible framework.”

Second, coach capability was viewed as central to successful implementation. Stakeholders emphasized that Lion Hearts delivery would require more than general coaching experience. Coaches would need to be comfortable engaging young children, adapting activities in real time, managing mother–child dyads safely, and creating sessions that were both engaging for children and meaningful for mothers. One participant summarized this requirement as, “You need the right coach […] someone who can pivot and be creative.” Stakeholders also emphasized the importance of prioritizing children’s enjoyment, with one participant noting, “For kids, it has to be fun first.”

Third, compatibility with existing affiliate operations emerged as an important consideration for adoption and continued delivery. Stakeholders identified scheduling, staffing, available space and equipment, and the characteristics of the affiliate population as factors that could influence whether Lion Hearts could be incorporated into routine operations. As one owner-coach explained, “This only works if it fits the gym’s space, schedule, and people.” Stakeholders therefore emphasized adapting implementation procedures to local conditions while preserving the intervention’s core components.

Stakeholders also identified several potential implementation assets. These included existing coaching expertise, adaptable affiliate environments, established relationships between coaches and members, access to fitness equipment, and prior experience delivering group-based programming. To strengthen traceability from stakeholder input to the implementation package, Table 2 links each overarching consideration and associated determinants to exemplar quotations, the co-design session in which the perspective was expressed, resulting implementation decisions, and refinements made following the second session. Participant identifiers are not reported because the small, geographically described sample could increase the risk of participant identification. Together, the identified needs, assets, barriers, and facilitators informed the implementation objectives and determinant-specific change objectives developed in Task 2.

### 3.3 Task 2: implementation outcomes, performance objectives, determinants, and change objectives

The Task 1 findings were translated into stakeholder-specific performance objectives describing the observable actions required for successful adoption and implementation. For affiliate owners, these objectives included committing to offer Lion Hearts, identifying appropriate coaches, allocating necessary resources, establishing feasible schedules, promoting the program to eligible families, and integrating the program into existing affiliate operations.

For coaches, performance objectives included completing the necessary program preparation and training, delivering the core intervention components, adapting activities for mother–child dyads, maintaining participant safety, managing class flow, facilitating engagement, and monitoring adherence to the intervention’s core components.

Determinants of these performance objectives included perceived feasibility, compatibility with existing operations, staffing capacity, scheduling constraints, competing demands, knowledge, behavioral capability, self-efficacy, perceived delivery complexity, and awareness of the intervention’s core components (Table 3). For each determinant, the research team specified what would need to change for affiliate owners and coaches to accomplish the associated performance objective. For example, affiliate owners would need to perceive Lion Hearts as feasible and compatible with their existing operations, whereas coaches would need sufficient knowledge, skills, confidence, and practical support to adapt and deliver the program as intended. Prospective implementation outcomes were specified as reach, adoption, implementation fidelity, feasibility, acceptability, and maintenance potential. These Task 2 outputs provided the basis for selecting theoretical change methods and designing the implementation strategies presented in Task 3.

**Table 3:** Implementation mapping matrix linking performance objectives, determinants, strategies, mechanisms, and outcomes for Lion Hearts.

Stakeholder & performance objective	Determinant	Theoretical change method	Implementation strategy & Lion Hearts application	Hypothesized mechanism	Proposed implementation outcome
Affiliate owner					
Commit to offering Lion Hearts and allocate necessary resources	Perceived feasibility; compatibility with existing operations	Anticipated outcomes; persuasive communication; tailoring	Assess readiness and identify barriers: Affiliate readiness/planning guide addressing scheduling, staffing, space, and equipment	Increased perceived compatibility and reduced anticipated implementation burden	Adoption; feasibility
Integrate Lion Hearts into affiliate schedules and workflows	Scheduling constraints; staffing capacity; competing demands	Action planning; problem solving; tailoring	Develop implementation plan: Scheduling and staffing framework	Increased compatibility with existing affiliate operations	Adoption; feasibility; maintenance
Coach					
Prepare to deliver core Lion Hearts components	Knowledge; behavioral capability; self-efficacy	Instruction; modeling; guided practice	Train and educate implementers: Coach implementation guide and training materials	Increased knowledge, skills, and confidence for delivery	Implementation (fidelity); feasibility
Deliver mother–child sessions while maintaining core components	Self-efficacy; perceived complexity	Guided practice; mastery experiences; feedback	Provide structured implementation supports: Session templates and movement-scaling guidance	Reduced perceived delivery complexity and increased coach capability	Implementation (fidelity); feasibility
Manage mothers and young children safely and efficiently	Class-flow demands; space and equipment constraints	Action planning; problem solving; tailoring	Restructure delivery procedures: Class-flow and equipment-organization guidance	Reduced logistical burden and increased compatibility with coaching practices	Feasibility; Implementation (fidelity)
Monitor delivery of core components	Knowledge of core components; competing coaching demands	Self-monitoring; feedback	Support self-monitoring of delivery: Fidelity checklist	Increased awareness of adherence to core components and identification of delivery deviations	Implementation (fidelity)

### 3.4 Task 4: implementation materials and delivery supports

Task 4 resulted in a preliminary implementation package designed to support adoption and delivery of Lion Hearts. The package included an affiliate readiness and planning guide, a scheduling and staffing framework, coach implementation and training materials, structured session templates, movement-scaling and safety guidance, class-flow and equipment-organization resources, fidelity-monitoring tools, family workbooks, and goal-setting resources.

During the second co-design session, affiliate owner-coaches reviewed the preliminary materials for anticipated usability, clarity, and compatibility with typical affiliate workflows. Their feedback reinforced the need for implementation supports that provided sufficient structure to protect the intervention’s core components without restricting coaches’ ability to respond to child behavior, differences in participant abilities, equipment availability, and changing class conditions. Feedback from the second co-design session resulted in several specific revisions to the preliminary implementation package. Session templates were revised to provide greater flexibility for responding to child behavior, differing abilities, interruptions, and changing class conditions. The materials were clarified to distinguish core intervention components from elements that coaches could adapt to local circumstances. Coach-facing resources were expanded to include additional movement-scaling examples, developmentally appropriate engagement strategies, and guidance for managing mother–child classes safely and efficiently. Affiliate-readiness materials were also refined to address local scheduling, staffing, space, equipment, and member-population considerations. These changes and their links to the original stakeholder input are summarized above in Table 2.

### 3.5 Task 5: prospective implementation evaluation plan

Task 5 produced a prospective mixed-methods plan for evaluating implementation during subsequent pilot delivery of Lion Hearts. The evaluation plan was organized using the RE-AIM framework and included Reach, Adoption, Implementation, and Maintenance, with Effectiveness assessed separately through participant-level health and behavioral outcomes. Feasibility and acceptability were included as complementary implementation outcomes to assess the practicality and perceived fit of Lion Hearts within community affiliate settings.

For each outcome, indicators, data sources, and assessment time points were specified prospectively. Reach will characterize the number, proportion, and characteristics of eligible mother–child dyads who enroll, participate, and remain in the program. Adoption will assess uptake among eligible affiliates and coaches, including reasons for participation or nonparticipation. Implementation will examine fidelity to core components, dose delivered, adaptations, and protocol deviations.

Feasibility and acceptability will capture affiliate owner, coach, and participant experiences with program delivery and the implementation supports. Because the current evaluation plan assesses intention to continue at the end of the intervention rather than longer-term institutionalization, this outcome is characterized as maintenance potential. Maintenance potential will include owner-coach intentions to continue offering Lion Hearts, its anticipated integration into routine affiliate operations, and the resources perceived as necessary for continued delivery.

Participant-level maternal, child, and family health and behavioral outcomes will be examined separately as preliminary indicators of intervention Effectiveness. This distinction will support assessment of whether subsequent findings reflect the effects of Lion Hearts, the success of its implementation, or both. Recipient perspectives will be incorporated during pilot implementation through interviews with mothers and age-appropriate feedback from children. These data will assess the perceived fit, accessibility, burden, and acceptability of Lion Hearts and identify recommended adaptations to program content and delivery. Recruitment, participation, and retention will also be examined in relation to socioeconomic and access-related factors, including program and membership costs, transportation, scheduling, cultural fit, disability-related accessibility, and prior affiliate membership. Implementation costs and participant-reported financial or logistical barriers will be documented to inform future decisions regarding equitable reach, adoption, and scalability. Table 4 summarizes the prospective evaluation plan. Because these outcomes have not yet been assessed, the plan provides a framework for empirically testing and refining the implementation strategies and hypothesized pathways developed through Implementation Mapping Tasks 1–4.

**Table 4:** Prospective evaluation of Lion Hearts implementation outcomes.

Outcome	What will be evaluated	Key indicators	Data source	Timing
Reach	Enrollment, attendance, and retention of eligible mother–child dyads	Enrolled eligible dyads ÷ all eligible dyads identified through screening. Compare enrolled and non-enrolled dyads on available demographic, geographic, membership, and baseline characteristics.	Screening, enrollment, attendance, and retention records; recruitment through post-intervention	Recruit planned sample; ≥80% participant retention; ≥75% mean session attendance
Adoption—affiliate	Uptake among eligible affiliates	Affiliates agreeing to deliver Lion Hearts ÷ eligible affiliates approached. Compare adopting and non-adopting affiliates by region, urban/rural context, size, staffing, and prior family/youth programming.	Affiliate recruitment records and owner feedback; pre-implementation	Report proportion adopting and reasons for non-adoption
Adoption—coach	Uptake among eligible implementation agents	Coaches agreeing to deliver Lion Hearts ÷ eligible coaches invited. Compare participating and nonparticipating coaches on available experience and credential characteristics.	Coach recruitment and training records; pre-implementation	At least one trained coach available at each adopting affiliate
Implementation	Fidelity to core components, dose delivered, adaptations, protocol deviations, and costs	Number of core components delivered ÷ number expected; number of sessions delivered ÷ number planned	Fidelity checklists, session records, and observations; throughout delivery	≥80% of core components delivered and ≥80% of planned sessions completed
Feasibility	Practicality of implementing Lion Hearts within affiliate settings	Owner, coach, and mother perceptions; scheduling, staffing, equipment, class flow, and usability	Feasibility of Intervention Measure and qualitative interviews; mid- and post-intervention	Mean FIM score ≥4 of 5
Acceptability	Stakeholder satisfaction, burden, and perceived fit	Owner, coach, and mother perceptions	Acceptability of Intervention Measure and qualitative interviews; mid- and post-intervention	Mean AIM score ≥4 of 5
Maintenance potential	Intention and perceived capacity to continue Lion Hearts after the pilot	Proportion of owners and coaches reporting an intention to continue; resources and adaptations required	Owner-coach surveys and interviews; post-intervention	Majority of owners and coaches report that continued delivery is likely or very likely

Outcomes are specified prospectively for evaluation during subsequent pilot implementation.

### 3.6 Integration of implementation mapping outputs

The outputs from Implementation Mapping Tasks 1–5 were integrated into an implementation logic model describing the hypothesized pathway from implementation determinants to implementation and intervention outcomes. As shown in Figure 3, the model specifies how stakeholder-informed implementation strategies are expected to address identified determinants through proposed implementation mechanisms. These mechanisms are hypothesized to influence adoption, feasibility, acceptability, implementation fidelity, and maintenance potential.

**Figure 3:**
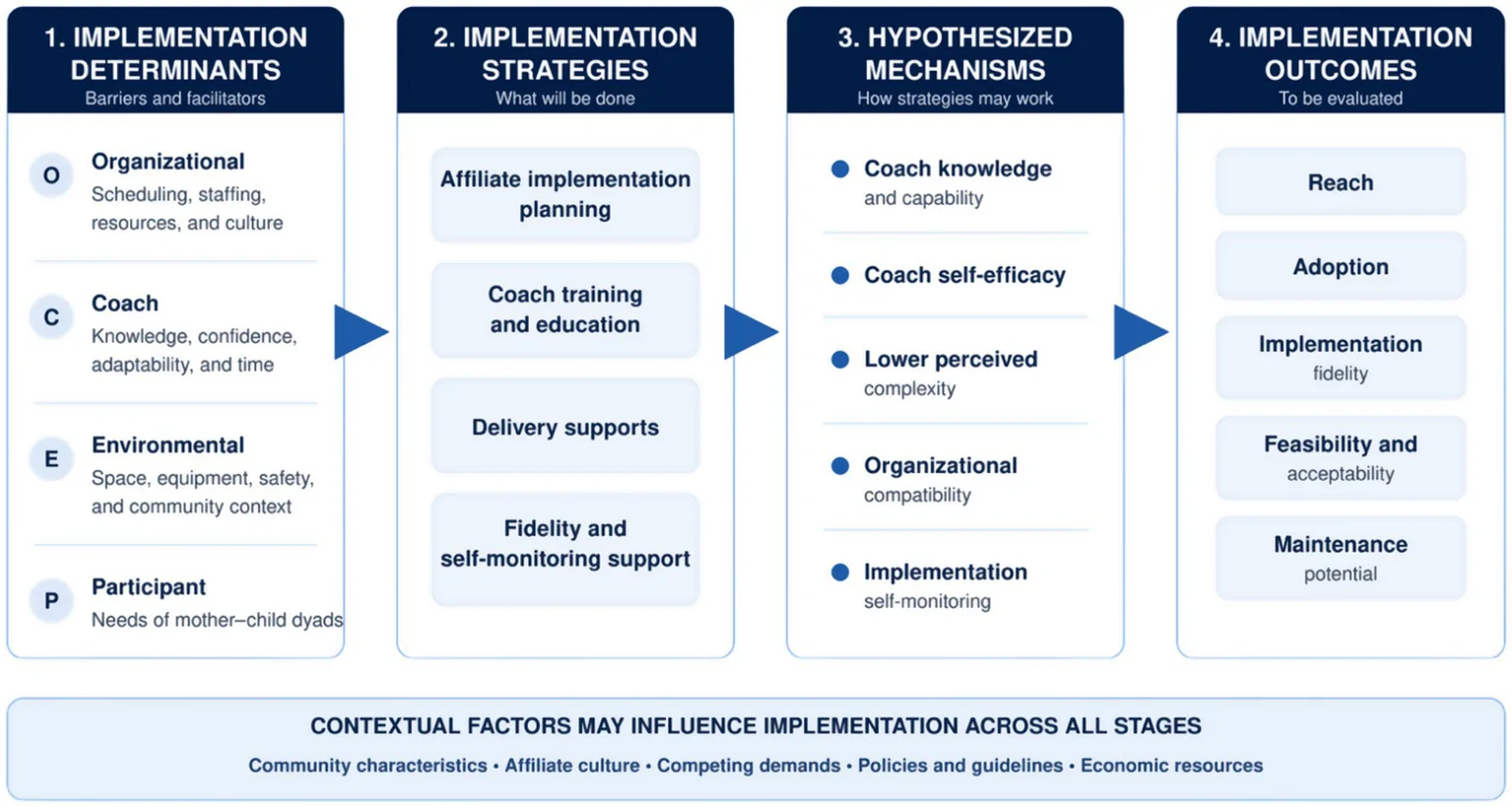
Implementation logic model.

Successful implementation is, in turn, expected to support delivery of the Lion Hearts intervention and contribute to participant-level maternal, child, and family health outcomes. The proposed pathways are prospective and will be examined and refined during subsequent pilot implementation.

## 4 Discussion

### 4.1 Principal findings

This study applied the Implementation Mapping framework to systematically develop implementation strategies and delivery supports for Lion Hearts, a family-based physical activity intervention intended for delivery within community CrossFit affiliates. Through engagement with experienced affiliate owner-coaches, three central implementation considerations emerged: the need to balance structure with flexibility during mother–child sessions, the importance of coach capability for managing and adapting family-based delivery, and the need for compatibility with existing affiliate operations. These findings informed the prospective mapping of stakeholder-specific performance objectives and determinants to theoretical change methods, implementation strategies, hypothesized mechanisms, and implementation outcomes. The resulting implementation supports included affiliate planning resources, coach training materials, session templates, movement-scaling and class-flow guidance, and fidelity tools.

Importantly, the present study represents an implementation-planning phase rather than an evaluation of implementation effectiveness. The strategies and mechanisms identified through Implementation Mapping have not yet been empirically tested for their effects on adoption, feasibility, fidelity, or maintenance. Rather, this work provides a theoretically and stakeholder-informed implementation package and a set of explicit, testable hypotheses for subsequent pilot implementation. This approach is consistent with implementation science recommendations to identify multilevel determinants and select strategies that explicitly address barriers and facilitators within the intended delivery context (43, 44).

A major contribution of this work is the extension of Intervention Mapping beyond intervention development to include structured implementation planning. While many community-based physical activity interventions demonstrate efficacy under controlled conditions, relatively few explicitly address how programs will be adopted, delivered, and sustained in practice (45). This gap is reflected across the physical activity literature, where implementation challenges frequently contribute to reduced reach, inconsistent delivery, and limited sustainability despite promising intervention outcomes (45, 46). By considering implementation during intervention development, the present study specifies how Lion Hearts could be integrated within community fitness settings and identifies the organizational and implementer-level conditions that warrant evaluation during pilot delivery. This proactive approach aligns with growing emphasis on incorporating implementation considerations earlier in intervention development rather than addressing barriers only after effectiveness has been established (29, 31). Moreover, studies applying Implementation Mapping have demonstrated its utility for systematically linking determinants, stakeholder behaviors, and implementation strategies and for articulating hypothesized mechanisms that can subsequently be tested (31).

### 4.2 Importance of community fitness settings

Community fitness organizations represent a promising but underutilized setting for family-based health promotion. Community-based exercise settings have been identified as important venues for translating evidence-based physical activity interventions into sustainable real-world practice because they provide existing infrastructure, established social networks, and opportunities for ongoing participation beyond the duration of a research study (32, 47). CrossFit affiliates may be particularly well positioned for family-based interventions due to their emphasis on community, supportive coaching, scalability, and adaptability to varying fitness levels (6, 48). Stakeholder findings in the present study further identified programming flexibility, social connection, and shared mother–child participation as potential strengths of this delivery setting.

However, the findings also demonstrate that the presence of existing fitness infrastructure does not necessarily translate into implementation readiness. Owner-coaches identified practical challenges related to scheduling, staffing, equipment and space, and maintaining child engagement. They also emphasized that implementation would need to accommodate variation across affiliates rather than rely on a single prescriptive delivery model. This tension between standardization and adaptability was particularly salient: stakeholders wanted sufficient structure to support delivery of core intervention components while retaining flexibility to respond to child behavior, class disruptions, available resources, and local workflows.

These findings are consistent with implementation science research demonstrating that organizational and logistical barriers can substantially influence intervention adoption, implementation quality, and sustainability (43). Similar challenges have been reported across community-based physical activity programs, where staffing capacity, competing demands, resource constraints, and participant attendance are among the most frequently cited barriers to successful implementation (49). The implementation supports developed in the present study—including affiliate planning resources, coach training, adaptable session templates, class-flow guidance, and fidelity monitoring—were designed specifically in response to these anticipated barriers. Whether these supports sufficiently address the identified determinants and facilitate implementation remains an empirical question for subsequent pilot testing.

### 4.3 Value of co-design in implementation planning

A notable strength of this study was the use of co-design methods to engage affiliate owner-coaches throughout the implementation planning process. Their dual experience with organizational decision-making and frontline coaching provided practice-based perspectives on both adoption and delivery. Their perspectives informed not only the identification of implementation determinants but also the development of concrete implementation supports related to scheduling, staffing, coach preparation, class flow, movement adaptation, and fidelity.

The transcript analysis also demonstrated how stakeholder input altered the practical form of the implementation package. For example, stakeholders emphasized that rigid programming would be poorly suited to the unpredictable nature of classes involving young children and instead favored a structured but adaptable framework. Similarly, their emphasis on coach creativity, comfort with children, and ability to respond to disruptions informed the development of coach-facing training and delivery supports. These findings illustrate the value of co-design not simply for identifying barriers, but for translating those barriers into contextually relevant implementation strategies and materials.

This approach is consistent with contemporary implementation science recommendations emphasizing stakeholder engagement during implementation planning (31, 34). Previous research has demonstrated that stakeholder involvement can improve the contextual relevance and compatibility of intervention and implementation approaches (35). However, stakeholder involvement alone does not establish acceptability, feasibility, implementation fidelity, or sustainability. These outcomes remain to be evaluated prospectively during pilot implementation. Accordingly, the present co-design process should be understood as an initial, context-specific implementation planning exercise that generated strategies and hypotheses for testing, rather than evidence that Lion Hearts is implementation-ready or scalable across affiliates.

### 4.4 Implications for future pilot testing

The implementation products developed through this process provide a foundation for prospective pilot testing of Lion Hearts and its implementation approach. The resulting package provides structured but adaptable implementation supports intended to preserve core intervention components while accommodating variation in affiliate staffing, scheduling, space, equipment, and class workflows. Whether these supports are feasible, acceptable, and sufficient to support consistent delivery across affiliate contexts remains to be empirically evaluated.

Future pilot studies should test the hypothesized pathways specified in the Implementation Mapping matrix and implementation logic model, including whether the selected strategies influence proposed mechanisms such as coach knowledge and behavioral capability, self-efficacy, perceived complexity, organizational compatibility, and implementation self-monitoring. These mechanisms can then be examined in relation to the prospective implementation outcomes specified in Table 4, including reach, adoption, implementation fidelity, feasibility, acceptability, and maintenance. Examining these pathways will help determine not only whether implementation is successful, but also how and under what contextual conditions the implementation strategies operate.

In parallel, future research should examine preliminary intervention effectiveness, including maternal physical activity and cardiovascular health indicators, child physical activity and movement-related outcomes, and family health behaviors. Simultaneous assessment of implementation and participant-level outcomes will help distinguish challenges related to intervention effectiveness from those related to intervention delivery. Findings from this pilot phase can subsequently inform refinement of the implementation package and determine whether evaluation across a larger and more diverse sample of affiliates is warranted.

### 4.5 Strengths and limitations

This study has several strengths. First, it employed a systematic and theory-informed Implementation Mapping approach to guide implementation planning and explicitly linked stakeholder-specific performance objectives, determinants, theoretical change methods, implementation strategies, hypothesized mechanisms, and prospective implementation outcomes. Second, stakeholder input was collected through co-design sessions that were audio-recorded and transcribed verbatim, with both transcripts independently reviewed and coded by three members of the research team using structured matrices. This approach provided a transparent process for translating stakeholder input into implementation strategies and practical delivery supports. Third, the inclusion of experienced affiliate owner-coaches provided practice-based perspectives on both organizational adoption and frontline delivery within community fitness settings. Finally, the development of a prospective implementation evaluation plan provides an explicit framework for empirically testing the proposed strategies and mechanisms during subsequent pilot implementation.

Several limitations should also be acknowledged. The implementation planning process involved seven affiliate owner-coaches recruited from a limited geographic region. The small, context-specific sample was intended to support formative implementation planning rather than thematic saturation or comprehensive characterization of implementation determinants across the broader CrossFit affiliate population. Accordingly, the determinants and strategies identified here may not capture the range of implementation conditions present across affiliates with different populations, organizational structures, resources, or geographic contexts. Additionally, because participants simultaneously served as affiliate owners and coaches, the study could not independently distinguish organizational adopter perspectives from frontline implementer perspectives. Future work should engage a broader range of stakeholders, including affiliate owners and coaches serving in distinct roles and individuals from more diverse affiliate contexts. Lastly, the implementation-planning sessions included affiliate owners and coaches but did not directly include mothers or children. Although mothers contributed to earlier intervention-development activities, their perspectives on the proposed implementation procedures and children’s perspectives remain important. Accordingly, the pilot evaluation will incorporate interviews with mothers and age-appropriate feedback from children and will examine affordability, transportation, cultural fit, accessibility, and socioeconomic diversity as potential influences on reach, adoption, and scalability.

The present implementation-planning phase also primarily captured the perspectives of those responsible for organizational adoption and program delivery and did not directly include mothers or children in these co-design sessions. Mothers contributed to the earlier Intervention Mapping stages that informed development of the Lion Hearts intervention (32); however, their perspectives on the proposed implementation strategies and delivery supports, as well as those of participating children, remain to be evaluated during pilot implementation. Finally, this study focused on prospective implementation planning and did not empirically evaluate the proposed implementation strategies, hypothesized mechanisms, or implementation and intervention outcomes. Thus, the present findings should not be interpreted as evidence of implementation feasibility, fidelity, effectiveness, sustainability, or scalability. These outcomes require prospective evaluation during subsequent pilot implementation.

## 5 Conclusion

Implementation Mapping provided a systematic approach for integrating implementation planning into the development of Lion Hearts, a family-based physical activity intervention intended for delivery within community CrossFit affiliates. Through stakeholder-informed planning, key implementation determinants were identified and systematically linked to stakeholder-specific performance objectives, theoretical change methods, implementation strategies, hypothesized mechanisms, and prospective implementation outcomes. These linkages informed the development of practical implementation supports tailored to anticipated barriers within community affiliate settings.

The resulting implementation package and evaluation plan provide a foundation for prospective pilot testing rather than evidence of implementation readiness or effectiveness. Future evaluation will determine whether the proposed strategies influence their hypothesized mechanisms and support reach, adoption, feasibility, acceptability, implementation fidelity, and maintenance in practice. More broadly, this work illustrates the value of incorporating structured implementation planning during the development of family-based physical activity interventions and provides a transparent approach for generating implementation strategies that can be empirically tested and refined within real-world community settings.

## Data Availability

The original contributions presented in the study are included in the article/Supplementary material, further inquiries can be directed to the corresponding author.
