# Supplementary material for: Lion Hearts: applying implementation mapping to plan the delivery of a family-based physical activity intervention in community CrossFit affiliates
Source: Front Public Health. 2026 Oct 6;14:1970725. doi: 10.3389/fpubh.2026.1970725 (PMC13680431; doi:10.3389/fpubh.2026.1970725)
Supplement: Supplementary file 1 [file Table_1.DOCX]

**Supplementary File 1. Co-Design Session Prompts**

Session 1: Implementation Needs and Assets Assessment

1. What would affiliate owners and coaches need to do to adopt and deliver Lion Hearts?
2. What factors could facilitate or impede adoption and delivery?
3. What challenges might arise when coaching mothers and young children together?
4. What training, resources, or delivery supports would coaches need?
5. How might scheduling, staffing, space, equipment, safety, and class flow affect implementation?
6. What factors could influence participant recruitment, engagement, attendance, and retention?

Session 2: Review and Refinement of Implementation Materials

1. How clear, useful, and practical are the proposed materials?
2. Do the materials provide an appropriate balance between structure and flexibility?
3. How well do the proposed procedures fit typical affiliate workflows?
4. What changes are needed to address coaching, safety, movement-scaling, equipment, or class-management concerns?
5. Which components should remain consistent, and which should be adaptable across affiliates?
6. What additional resources would support successful and continued delivery?
