# Supplementary material for: Lion Hearts: applying implementation mapping to plan the delivery of a family-based physical activity intervention in community CrossFit affiliates
Source: Front Public Health. 2026 Oct 6;14:1970725. doi: 10.3389/fpubh.2026.1970725 (PMC13680431; doi:10.3389/fpubh.2026.1970725)
Supplement: Supplementary file 2 [file Table_2.DOCX]

**Supplementary Table S2. Prospective Specification of Lion Hearts Implementation Strategies**

| **Lion Hearts implementation strategy** | **Actor** | **Action** | **Action target** | **Planned timing** | **Planned dose/intensity** | **Intended implementation outcome** |
| --- | --- | --- | --- | --- | --- | --- |
| Affiliate readiness assessment and barrier identification | Research team and affiliate owner | Review anticipated barriers and available resources related to scheduling, staffing, space, equipment, and participant recruitment | Organizational readiness, perceived feasibility, and compatibility | Before the affiliate commits to program delivery | One pre-implementation readiness review, with follow-up as needed | Adoption; feasibility |
| Affiliate implementation planning | Affiliate owner, supported by the research team | Develop an affiliate-specific plan addressing scheduling, staffing, space, equipment, recruitment, and permitted adaptations | Organizational planning, resource allocation, and workflow compatibility | Before participant recruitment and program delivery | One initial implementation plan, revised as needed before launch | Adoption; feasibility; maintenance potential |
| Coach training and implementation guide | Research team | Provide instruction and guidance on core components, mother–child delivery, movement scaling, safety, participant engagement, and fidelity expectations | Coach knowledge, behavioral capability, and self-efficacy | Before the first Lion Hearts session | One pre-implementation training session and continued access to the coach guide | Implementation fidelity; feasibility |
| Adaptable session templates and movement-scaling guidance | Research team provides materials; coaches apply them | Provide structured session plans, scaling options, and guidance distinguishing core components from adaptable elements | Coach preparation, perceived complexity, and ability to adapt delivery | Provided before implementation and reviewed before each session | One template for every Lion Hearts session | Implementation fidelity; feasibility |
| Class-flow and equipment-organization guidance | Affiliate owner and coach | Plan participant flow, activity transitions, equipment placement, and use of available space | Logistical burden, safety, class flow, and compatibility with affiliate operations | Initially before implementation and reconsidered before individual sessions | One initial class-flow plan with session-specific adjustments as needed | Feasibility; implementation fidelity |
| Fidelity self-monitoring checklist | Coach | Document delivery of core components, adaptations, and protocol deviations | Coach awareness of adherence and identification of delivery challenges | Immediately following each Lion Hearts session | One checklist after every session | Implementation fidelity |
